# Development and validation of a nomogram model based on pretreatment ultrasound and contrast-enhanced ultrasound to predict the efficacy of neoadjuvant chemotherapy in patients with borderline resectable or locally advanced pancreatic cancer

**DOI:** 10.1186/s40644-024-00662-2

**Published:** 2024-01-20

**Authors:** Xiaoyi Yan, Xianshui Fu, Yang Gui, Xueqi Chen, Yuejuan Cheng, Menghua Dai, Weibin Wang, Mengsu Xiao, Li Tan, Jing Zhang, Yuming Shao, Huanyu Wang, Xiaoyan Chang, Ke Lv

**Affiliations:** 1 Department of Ultrasound, Peking Union Medical College Hospital, Peking Union Medical College, Chinese Academy of Medical Sciences, Beijing, 100730 China; 2 Department of Ultrasound, No.304 Hospital of Chinese PLA, Beijing, 100037 China; 3 Department of Medical Oncology, Peking Union Medical College Hospital, Peking Union Medical College, Chinese Academy of Medical Sciences, Beijing, 100730 China; 4 Department of General Surgery, Peking Union Medical College Hospital, Peking Union Medical College, Chinese Academy of Medical Sciences, Beijing, 100730 China; 5 Department of Pathology, Peking Union Medical College Hospital, Peking Union Medical College, Chinese Academy of Medical Sciences, Beijing, 100730 China

## Abstract

**Objectives:**

To develop a nomogram using pretreatment ultrasound (US) and contrast-enhanced ultrasound (CEUS) to predict the clinical response of neoadjuvant chemotherapy (NAC) in patients with borderline resectable pancreatic cancer (BRPC) or locally advanced pancreatic cancer (LAPC).

**Methods:**

A total of 111 patients with pancreatic ductal adenocarcinoma (PDAC) treated with NAC between October 2017 and February 2022 were retrospectively enrolled. The patients were randomly divided (7:3) into training and validation cohorts. The pretreatment US and CEUS features were reviewed. Univariate and multivariate logistic regression analyses were used to determine the independent predictors of clinical response in the training cohort. Then a prediction nomogram model based on the independent predictors was constructed. The area under the curve (AUC), calibration plot, C-index and decision curve analysis (DCA) were used to assess the nomogram’s performance, calibration, discrimination and clinical benefit.

**Results:**

The multivariate logistic regression analysis showed that the taller-than-wide shape in the longitudinal plane (odds ratio [OR]:0.20, *p* = 0.01)**,** time from injection of contrast agent to peak enhancement (OR:3.64; *p* = 0.05) and Peak_tumor_/ Peak_normal_ (OR:1.51; *p* = 0.03) were independent predictors of clinical response to NAC. The predictive nomogram developed based on the above imaging features showed AUCs were 0.852 and 0.854 in the primary and validation cohorts, respectively. Good calibration was achieved in the training datasets, with C-index of 0.852. DCA verified the clinical usefulness of the nomogram.

**Conclusions:**

The nomogram based on pretreatment US and CEUS can effectively predict the clinical response of NAC in patients with BRPC and LAPC; it may help guide personalized treatment.

## Introduction

Pancreatic cancer is predicted to be the second leading cause of cancer-related death by 2030 [1], in which pancreatic ductal adenocarcinoma (PDAC) accounts for the majority [2]. Due to vague symptoms and early involvement of large blood vessels, surgical treatment is only feasible for 20% of patients [3]. For patients without metastatic disease but showing borderline resectable pancreatic cancer (BRPC) or locally advanced pancreatic cancer (LAPC), neoadjuvant chemotherapy (NAC) may improve the rate of a margin negative (R0) resection and the overall survival (OS) [4]. However, while some patients derive significant clinical benefit, most patients present the lack of a major tumor response to NAC [5]. Inadequate tumor response may facilitate tumor progression, obviating the opportunity to completely resect the tumor [2, 6]. Several meta-analyses have reported the resection rate was lower in neoadjuvant therapy (NAT) group than upfront surgery group [7, 8]. Jung et al. [7] thought that lacking reliable criteria to select patients who are suitable to undergo surgical resection and the low rate to complete chemotherapy cycles due to various adverse effects from anti-cancer agents may be associated with the above results. In this case, effective radiographic and clinical data are needed to early discriminate the responders from non-responders and help guide the initial therapeutic management.

Given that traditional cross-sectional imaging modalities such as CT and/or MRI poorly predict response, metabolic and functional imaging parameters such as standardized uptake values from PET, apparent diffusion coefficient from diffusion-weighted imaging and quantitative pharmacokinetics of contrast-enhanced endoscopic ultrasound (CE-EUS) have been explored and confirmed to be associated with treatment response and survival [9–11]. Emori et al. [11] reported that patients with rich vascularity at both the arterial and venous phases in CE-EUS were considered to have a better response to gemcitabine and nab-paclitaxel. Tanaka et al. [12] reported detection of avascular areas by CE-EUS after chemotherapy may predict long-term survival of patients with PDAC.

Similar to CE-EUS, contrast-enhanced ultrasound (CEUS) is a promising tool for its ability to provide microvascular information about organs, which can help us to better understand angiogenesis across a variety of types of cancer. In addition, it is non-invasive as well as non-radiative and was recommended to be used in liver diseases by the World Federation for Ultrasound in Medicine and Biology (WFUMB) [13]. Previous studies have confirmed that the CEUS enhancement and wash-out patterns can effectively differentiate the solid pancreatic tumors [14–17]. Huang et al. [16] reported that hypo-enhancement in all phases, hyper-enhancement/iso-enhancement followed by washout on CEUS, an ill-defined border, and a dilated main pancreatic duct were independent risk factors for malignant solid pancreatic lesions (MSPL). The nomogram based on deep learning CEUS and clinical factors could serve as a preoperative, noninvasive, and precise evaluation tool to differentiate aggressive and non-aggressive pancreatic neuroendocrine neoplasms [17]. CEUS can also provide insight into tumor viability, in which patients with iso-enhancement of PDAC in the arterial phase have a significant longer overall survival time than that with hypo-enhancement pattern [14]. The European Federation of Societies for Ultrasound in Medicine and Biology (EFSUMB) also recommended that CEUS can be used as a reliable tool for characterizing PDAC in solid pancreatic lesions detected on ultrasound. Now we hypothesize that CEUS can predict the treatment response of NAC in patients with BRPC or LAPC and aim to develop a predictive nomogram based on pretreatment ultrasound (US) and CEUS to early verify patients who may benefit from NAC.

## Materials and methods

### Patients

Between October 2017 and February 2022, a total of 111 consecutive patients diagnosed as PDAC and treated with NAC were retrospectively enrolled. The patients were randomly divided (7:3) into training and validation cohorts.

The main inclusion criteria were histologically proven PDAC (by EUS or US guided biopsy) and medical fitness for NAC. The exclusion criteria were: (1) Patients with progressive disease or patients refusing chemotherapy; (2) Patients with metastasis at final CT/MRI; (3) Patients without complete clinical data or ultrasound images; (4) patients who were CA 19–9 nonproducers (< 1U/mL).

### US and CEUS techniques

All scans were performed using either a Philips IU22 scanner or a Epic7(Philips Healthcare, Bothell, WA, USA) equipped with a C 5–1 probe. All patients were required to fast for at least eight hours before examination. With B-mode US, positions, echoes, sizes, borders, and blood flow of the pancreatic lesions were recorded. CEUS was then performed. The mechanical index (MI) for the CEUS examination was 0.07. The ultrasonic contrast agent was dissolved in 5 mL of saline according to the manufacturer’s instructions. A rapid bolus injection of 2.4 mL of the contrast agent was administered intravenously at the first examination, followed by 5 mL of saline solution; if repeated injection is required for observation, a dose of 1.2 mL contrast agent was injected. The patient was in a stationary position for continuous real-time observation of the dynamic perfusion process, the duration of which was not less than 2 min. The enhancement phases were divided into the arterial phase (0–30 s after injection) and the venous phase (31–120 s after injection). Still images and video clips from the US and CEUS examinations were digitally stored for further evaluation.

### Baseline US and CEUS image analysis

US images and CEUS videos were analyzed by two experienced radiologists (K.L and Y. G, with 20 and 10 years of experience in abdominal ultrasound, respectively) who were blinded to clinical and pathologic results.

The following characteristics of the tumors were evaluated: (1)US-based tumor size ( the maximum diameter of the tumor); (2) tumor location; (3) solid or cystic-solid tumors; (4) blood flow based on color doppler US; (5) main pancreatic duct (MPD) dilatation (> 4 mm); (6) lobulated tumor shape; (7) celiac artery involvement; (8) taller-than-wide shape in the longitudinal plane (anteroposterior diameter exceeding the suprainferior diameter); (9) taller-than-wide shape in the transverse plane (anteroposterior diameter exceeding the transverse diameter); (10) necrosis based on CEUS; (11) time from injection of contrast agent to peak enhancement (TTP); (12) CEUS enhancement patterns in the arterial phase and wash-out patterns in the venous phase: The enhancement of the lesion was classified as either iso-enhancement (equivalent to that of the surrounding pancreatic parenchyma) or hypo-enhancement (less than that of the surrounding pancreatic parenchyma) in the arterial phase and the washout patterns were classified into two types, including fast washout (the washout of the lesion faster than pancreatic parenchyma and slow washout (the washout of the lesion slower than pancreatic parenchyma).

Static images were extracted from the time points of peak enhancement of CEUS video clips. The quantitative analysis of the enhancement was obtained by Image J calculating the greyscale median (GSM; median of the frequency distribution of the grey levels of the pixels) of a region-of-interest (ROI) localized in the tumor and in the adjacent parenchyma. The two ROIs were selected avoiding the blood vessels and the necrotic areas and the ROI of the tumor covered the whole area as much as possible. Tumor /tissue ratio was then calculated and recorded as Peak_tumor_/Peak_normal_.

### NAC

Systemic chemotherapy consisted of nab-paclitaxel + gemcitabine (AG) and nab-paclitaxel + tegafur (AS) administered in either 4-week or 2-week intervals. Chemotherapy duration was determined by the response and tolerance to the treatment.

### Response to NAC

According to the published literature, clinical response in patients were evaluated based on the Response Evaluation Criteria in Solid Tumors (RECIST, version 1.1) combined with the changes of serum levels of cancer antigen 19–9 (CA 19–9) [18, 19].

Before initiation of the chemotherapy and after 4–6 cycles of treatment, MDCT was used to evaluate anatomic cancer staging. Changes were recorded based on the RECIST Version 1.1 [20]. Progressive disease (PD) was defined as present of metastatic lesions or an increase of 20% in the primary tumor’s largest dimension (with a minimum increase of 5 mm). A partial response (PR) was defined as a decrease in the largest dimension of the primary tumor by at least 30%. Stable disease (SD) was defined as insufficient increase or decrease in tumor size to qualify as PD or PR, respectively. A complete response (CR) refers to the total disappearance of the primary tumor.

Serum CA19-9 levels were detected before and after treatment. Serologic responders were defined according to the published literature [19, 21]. Baseline CA19-9 were classified as: (1) normal CA19-9 (≤ 34 U/mL); (2) elevated CA19-9 (> 34 U/mL). Post-chemotherapy CA19-9 response categories were as: (1) normal baseline CA19-9 staying normal; (2) normal baseline CA19-9 then elevated; (3) elevated baseline CA19-9 and staying elevated; (4) elevated baseline CA19-9 and normalized. Patients with normal CA19-9 post-chemotherapy, whether normal or elevated pre-chemotherapy were defined as “Optimal CA19-9 Response”. Patients who were CA 19–9 nonproducers (< 1U/mL) were excluded.

Based on the radiographic and serologic response metrics, combined response categories were determined. Patients with radiographic response of CR or PR、radiographic response of SD but with optimal CA19-9 response were considered of responders to NAC. Others were considered ineffective.

### Statistical analysis

Qualitative data were presented as percentages and absolute numbers, while quantitative data were presented as means ± standard deviations. Student’s t test or the Mann–Whitney U test was used for continuous variables, and the *x*^2^ or Fisher exact test was used for categorical variables. Univariate analysis and multivariate logistic regression analysis were used to determine the independent predictors of clinical response in the training cohort. Then a prediction nomogram model based on the independent predictors was constructed. Area under the curve (AUC) of the receiver operating characteristic (ROC) was used to evaluate the performance of the nomogram. The calibration plot, C-index and decision curve analysis (DCA) were used to assess the nomogram’s calibration, discrimination and clinical benefit.

Cohen’s kappa coefficient was used to evaluate the interobserver agreement in analysing the qualitative imaging features of PDAC. A κ value < 0.2 indicates poor agreement; 0.2–0.4 indicates fair agreement; 0.41–0.6 indicates moderate agreement; 0.61–0.8 indicates good agreement; and 0.81–1 indicates almost perfect agreement. We defined significance as *p* < 0.05, except for univariate logistic analysis where *p* < 0.1 was thought as significance. SPSS 26.0 (IBM Corp) and the open-source statistical environment R (version 4.6.2) were used for statistical analysis.

## Results

### Patients’ characteristics

**Fig. 1:**
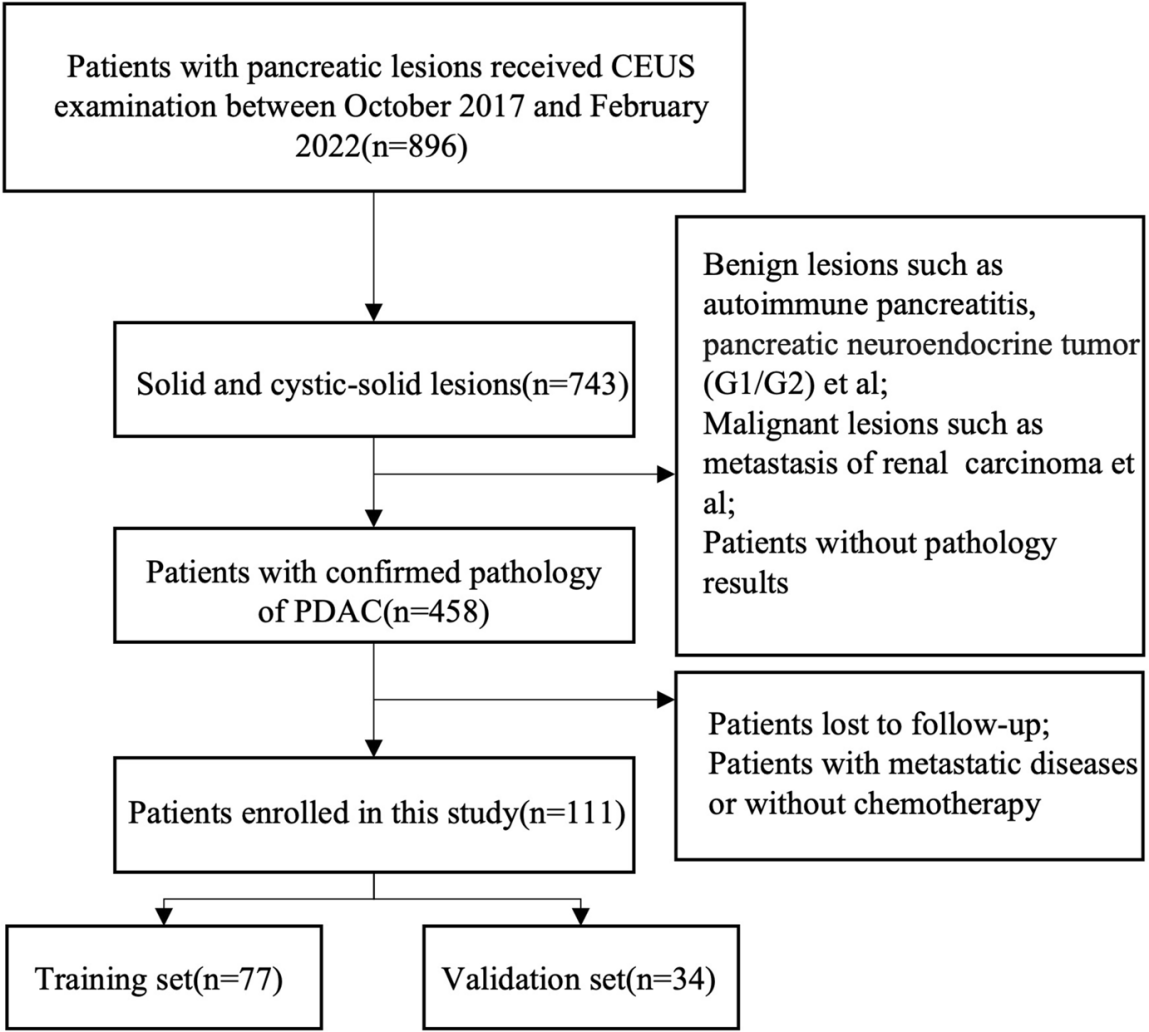
A patient selection flowchart

One hundred eleven eligible patients were enrolled in this study, of whom 77 were assigned to the training cohort and 34 to the validation cohort. The patient selection flowchart was shown in Fig. 1. Baseline characteristics were summarized in Table 1. There was significant difference in CA19-9 between the training and validation cohorts. After 4–6 cycles of NAC, 56(50.5%) patients were regarded as responders, the other 55 (49.5%) patients were regarded as non-responders. Eventually, 21.6% (24/111) patients underwent surgery after NAC. The overall surgical rate was 59.3% (16/27) in BRPC group while 9.5% (8/84) in LAPC group.

**Table 1:** Baseline characteristics of training and validation cohorts

	Training cohort	Validation cohort	*P* value
Age(year)	59.41 ± 1.52	60.44 ± 0.99	0.93
Sex (Male/Female)	29/27	31/24	0.63
CA19-9(U/mL)	437.32 ± 122.90	879.13 ± 184.70	0.05
Tumor size(cm, IQR)	4.98(1.95)	4.27(1.75)	0.27
Location			0.22
Head or neck	29	17	
Body or tail	48	17	
Tumor Stage (BRPC/LAPC)	18/59	9/25	0.73
Treatment (AG/AS)	41/36	18/16	0.98

*IQR* Interquartile range, *BRPC* Borderline resectable pancreatic cancer, *LAPC* Locally advanced pancreatic cancer, *AG* Nab-paclitaxel + gemcitabine, *AS* Nab-paclitaxel + tegafur

### Inter-observer reproducibility of feature extraction

The inter-observer reproducibility of feature extraction by the two radiologists was good, with ICCs ranging from 0.620 to 0.793. The controversial feature for analysis was negotiated by the two radiologists. A third reviewer was consulted when the consensus could not be reached.

### Comparison of baseline and imaging features in the training cohort

Baseline and imaging characteristics were compared between the responders and non-responders in the training set. The CA 19–9 level was higher among the non-responders than the responders. The group of non-responders have a larger mean tumor size than responders (*p* = 0.04) and the former have more lobulated and taller-than-wide shape in the longitudinal plane lesions (*p* = 0.02 and *p* < 0.01). As for the CEUS patterns, there were significant difference in the washout velocity of the venous phase (*p* = 0.01), TTP (*p* < 0.01) and Peak_tumor_/Peak_normal_ (*p* < 0.01). The two groups were comparable for lesion location (*p* = 0.93), nature (*p* = 0.72), MPD dilation (*p* = 0.36), celiac artery involvement (*p* = 0.28), taller-than-wide shape in the transverse plane (*p* = 0.28), presence of color doppler blood flow (*p* = 0.94), presence of necrosis (*p* = 0.08) and enhancement pattern in the arterial phase (*p* = 0.70). Details were shown in Table 2.

**Table 2:** Baseline and imaging characteristics of responders and non-responders in the training set

Imaging characteristics	Responders(34)	Non-responders(43)	*P* value
Age(year)	59.53 ± 1.60	61.16 ± 1.24	0.42
Sex (Male/Female)	17/17	26/17	0.36
CA19-9(U/mL)	512.68 ± 198.26	896.84 ± 216.89	0.05
Location			0.93
Head or neck	13	16	
Body or tail	21	27	
Tumor size(cm)	4.27 ± 0.22	4.83 ± 0.20	0.04
Nature			0.72
Solid	28	34	
Cystic-solid	6	9	
MPD dilation (> 4 mm)	12	11	0.36
Lobulated lesion shape	8	21	0.02
Celiac artery involvement	8	15	0.28
Taller-than-wide shape in the longitudinal plane	12	33	< 0.01
Taller-than-wide shape in the cross plane	8	15	0.28
Blood flow in color doppler	10	13	0.94
Necrosis	4	12	0.08
CEUS patterns			
Arterial phase			0.50
Iso-enhancement	20	22	
Hypo-enhancement	14	21	
Venous phase			0.01
Rapid washout	33	33	
Slow washout	1	10	
TTP			< 0.01
≤ 25 s	25	11	
> 25 s	9	32	
Peak_tumor_/Peak_normal_	0.63 ± 0.03	0.47 ± 0.03	< 0.01
Treatment			
AG	15	26	0.15
AS	19	17	

*MPD* Main pancreatic duct, *CEUS* Contrast-enhanced ultrasound, *TTP* Time from injection of contrast agent to peak enhancement, *Peak*_*tumor*_*/Peak*_*normal*_ Tumor/surrounding pancreatic parenchyma ratio of enhancement, *AG* Nab-paclitaxel + gemcitabine, *AS* Nab-paclitaxel + tegafur

### Independent predictors for tumor response

The results of univariate and multivariate analyses of related factors of treatment response were shown in Table 3. Among US and CEUS characteristics, smaller tumor size (*p* = 0.07), lobulated lesion shape (*p* = 0.03), taller-than-wide shape in the longitudinal plane (*p* < 0.001), presence of necrosis (*p* = 0.09), rapid washout pattern in the venous phase (*p* = 0.06), TTP (*p* < 0.001) and Peak_tumor_/Peak_normal_ (*p* = 0.001) were associated with tumor response in PDAC. Multivariate logistic regression was conducted based on the above factors. We found that taller-than-wide shape in the longitudinal section (*p* = 0.01), TTP (*p* = 0.05) and Peak_tumor_/Peak_normal_ (*p* = 0.01) were independent associated factors of tumor response.

**Table 3:** The univariate and multivariate analyses of treatment response in the training set

Imaging characteristics	Univariate analysis			Multivariate analysis		
	OR	95%CI	*P* value	OR	95%CI	*P* value
Location	0.38	0.38–2.42	0.93			
Tumor size (cm)	0.72	0.50–1.03	0.07	0.81	0.47–1.40	0.45
Nature	0.81	0.26–2.55	0.72			
MPD dilation (> 4 mm)	1.59	0.59–4.24	0.36			
Lobulated lesion shape	0.32	0.12–0.87	0.03	0.94	0.22–4.04	0.93
Celiac artery involvement	0.57	0.21–1.58	0.28			
Taller-than-wide shape in the longitudinal plane	0.17	0.06–0.45	< 0.001	0.20	0.05–0.72	0.01
Taller-than-wide shape in the cross plane	0.57	0.21–1.58	0.28			
Blood flow in color doppler	0.96	0.36–2.57	0.94			
Presence of necrosis	0.34	0.10–1.19	0.09	0.61	0.11–3.72	3.72
Arterial phase (Iso/hypo-enhancement)	1.36	0.55–3.38	0.50			
Venous phase (Rapid/slow washout)	0.33	0.11–1.03	0.06	0.10	0.01–1.11	0.06
TTP (≤ 25 s/ > 25 s)	8.08	2.90–22.51	< 0.001	3.64	1.00–13.41	0.05
Peak_tumor_/Peak_normal_	1.69	1.25–2.29	0.001	1.51	1.05–2.17	0.03
Treatment (AG/AS)	1.94	0.78–4.82	0.16			

*MPD* Main pancreatic duct, *CEUS* Contrast-enhanced ultrasound, *TTP* Time from injection of contrast agent to peak enhancement, *Peak*_*tumor*_*/Peak*_*normal*_ Tumor/surrounding pancreatic parenchyma ratio of enhancement, *AG* Nab-paclitaxel + gemcitabine, *AS* Nab-paclitaxel + tegafur

### Development and validation of nomogram

**Fig. 2:**
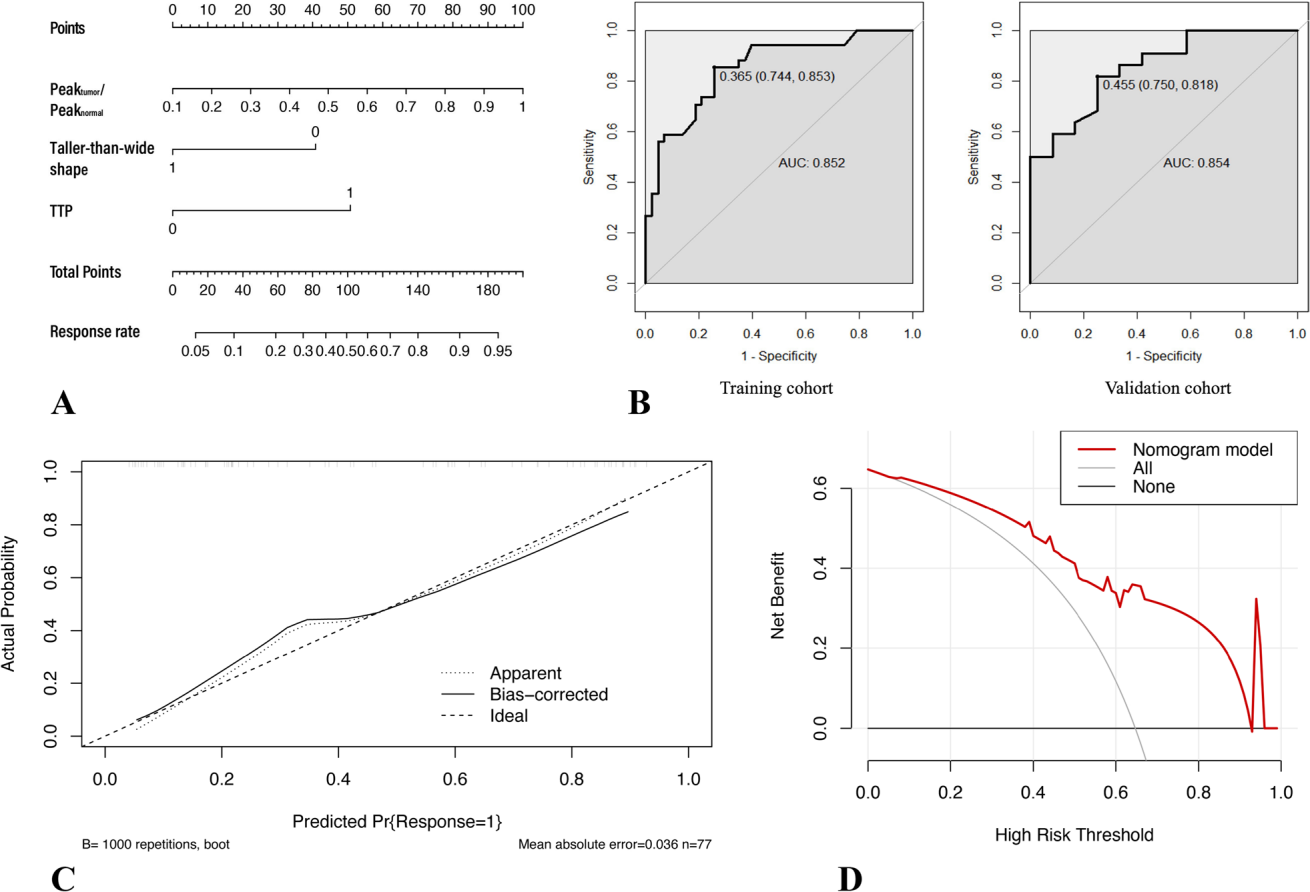
**A** Nomogram for predicting tumor response (To use the nomogram, an individual patient’s value is located on each variable axis, and a line is drawn upward to determine the number of points received for each variable value. The sum of these numbers is located on the total points axis, and a line is drawn downward to the risk axes to determine the probability of being “responders”) **B** ROC of the nomogram in the training and validation cohort **C** Calibration curve of the nomogram in the training cohort **D** Decision curve analysis for the nomogram. The y-axis measures the net benefit, x-axis indicates threshold probability. The red line represents the net benefit of the predictive nomogram

**Fig. 3:**
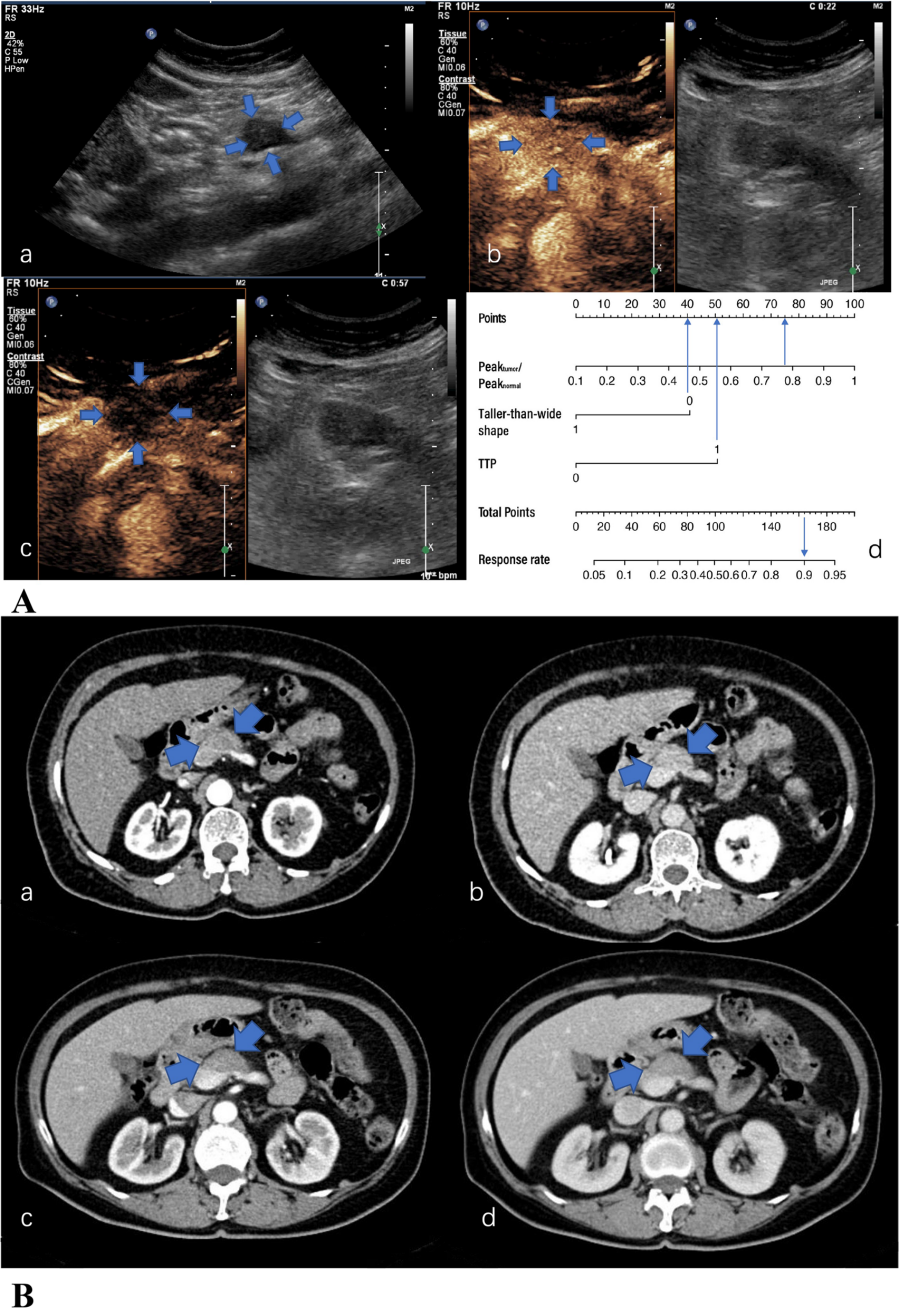
**A** US and CEUS of a 67-year-old woman with a 3.0 cm hypoechoic solid lesion in the head of the pancreas. a The lesion was not in a taller-than-wide shape in the longitudinal plane. b The TTP was 24 s and the Peak_tumor_/Peak_normal_ was 0.772 (in the transverse plane). c The lesion in the venous phase (in the transverse plane). d A total of 170 points were assigned to the lesion according to the nomogram, corresponding to an about 90% probability of “responders”. After 4 cycles of NAC, a radiographic response of SD was achieved. **B** The pre-treatment (a, b) and post-treatment (c, d) CT images of the case. CA 19–9 decreased from 50.8 to 20.3 U/mL

**Fig. 4:**
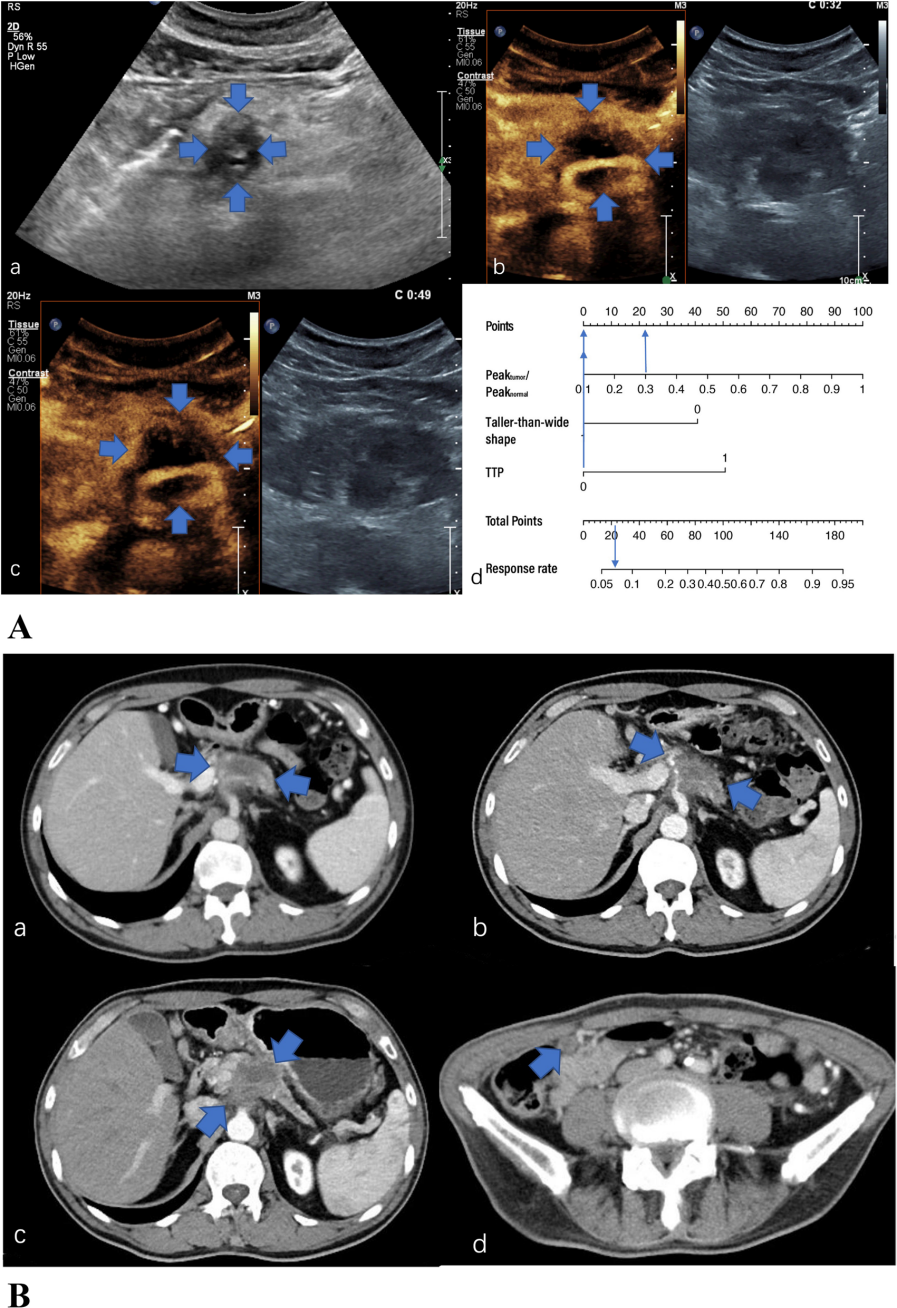
**A** US and CEUS of a 63-year-old man with a 4.1 cm hypoechoic solid lesion in the head of the pancreas. a The lesion was taller-than-wide in the longitudinal plane. b The TTP was 32 s and the Peak_tumor_/Peak_normal_ was 0.306 (in the transverse plane). c The lesion in the venous phase(in the transverse plane). d A total of 22 points were assigned to the lesion according to the nomogram, corresponding to an about 7% probability of “responders”. After 6 cycles of NAC, a radiographic response of PD (metastasis to paracolic lymph nodes) achieved. **B** The pre-treatment (a) and post-treatment (b, c, d) CT images of the case. The tumor was larger (b, c) and there was suspected paracolic lymph node metastasis (d) after 6 cycles of NAC.CA 19–9 decreased from 177.2 to 76.7U/mL

Based on the results of multivariate logistic regression analysis, a nomogram model was constructed. The discrimination efficacy was comparable between the training and validation sets, with AUCs of 0.852 (95%CI: 0.766–0.938) and 0.854 (95%CI:0.726–0.983), respectively. Calibration plot was conducted through bootstrapping with 1000 resamples. Good calibration was achieved for the nomogram in the training set, with C-index of 0.852. DCA was used to assess the utility of the predictive nomogram model by calculating the net benefit at various probability thresholds and it showed that the nomogram was a reliable clinical tool to predict the tumor response in patients treated with NAC (Fig. 2). In Figs. 3 and 4, two cases of combined response evaluated as responders and non-responders demonstrating the use of the prediction nomogram were presented.

## Discussion

The current guidelines from the National Comprehensive Cancer Network and American Society of Clinical Oncology recommend NAT followed by restaging and resection in PDAC patients who are possible candidates for surgical resection [22, 23]. Nevertheless, chemotherapy resistance and adverse effects of the anti-cancer drugs may make some patients miss the opportunity of surgery. Studies reported that the overall resection rate was lower in NAT group than upfront surgery group [7, 8]. Reliable diagnostic methods are urgently needed to accurately predict the tumor response, as well as to optimal therapeutic selection in patients that are suitable for NAC. Patient-derived organoids obtained from patients previously receiving cytotoxic chemotherapies are a promising technology to predict good clinical response to systemic chemotherapy in PDAC [24]. But the high cost and complicated technology may limit the generalization. In this study, we developed a predictive nomogram for “responders” of NAC based on the imaging features of pretreatment US and CEUS. The clinical chemotherapeutic response which was evaluated in a manner consistent with best practices, using both the well-validated imaging criteria (RECIST) and PDAC-specific tumor marker, CA 19–9 was selected as gold standard. The constructed nomogram could discriminate the responders from the non-responders effectively with an AUC of 0.852 in the training set and 0.854 in the test set.

CEUS is a real-time imaging technique that can visualize the microvascular perfusion of tumors non-invasively. Studies have reported CEUS can early predict treatment response in patients with various tumors who have received NAC [25–28]. Wan et al. [26] revealed that PEAK (the maximum intensity of the time-intensity curve during bolus transit), PEAK% and TTP% were significant independent predictors of pCR in breast cancer. Amdori et al. [28] found CEUS could be useful for detecting and quantifying dynamic changes in tumor vascularity of metastatic colorectal cancer. In PDAC, only one study reported that CEUS quantitative parameters of the responders such as rise time, wash-in area under the curve decreased significantly after NAC, which indicated CEUS might be a potential imaging method for non-invasive follow-up of early response [29]. However, most of the above studies were based on the relatively small sample sizes and need both pre-treatment and post-treatment CEUS images, which could not achieve an earlier prediction to guide NAC administration. Predictive models using only pre-treatment imaging techniques have been successfully developed. Li et al. [30] constructed a nomogram using related factors of pretreatment DCE-MRI to predict pCR after NAC in patients with triple-negative breast cancer, achieving an AUC of 0.84. A deep learning radiomics nomogram based on pretreatment contrast-enhanced CT images and clinical features showed satisfactory discrimination of good response to NAC in patients with locally advanced gastric cancer (AUC:0.829) [31]. To the best of our knowledge, this was the first study trying to predict response of NAC in PDAC patients using pretreatment US and CEUS features.

Our study found that patients with taller-than-wide shape lesions in the longitudinal plane were prone to be “non-responders” after NAC. The possible reasons may include: (1) A taller-than wide shape in US is commonly thought to reflect the anti-gravitational growth pattern across the normal tissue plane in malignant thyroid and breast nodules [32–34]. Similarly, lesions with taller-than-wide shape in PDAC may indicated the anti-gravitational growth pattern across the normal pancreas plane; (2) Pancreatic cancer is generally considered an extremely aggressive tumor with high invasion and metastatic propensity [6]. Most cases cannot achieve margin negative resection due to the involvement of major vascular structures such as superior mesenteric artery and celiac trunk at the time of diagnosis. PDAC lesions with taller-than-wide shape in the longitudinal plane in our study mainly grew infiltratively into the deep side of the tumor. We thought this kind of morphologic changes reflected the increased ability of tumor cells to proliferate to some extent, as well as the greater involvement of the major blood vessels.

Prominent pathological features of PDAC include an expansive, desmoplastic stroma characterized by high mechanical stiffness and low microvascular intensity (MVD) [6, 35]. The high mechanical pressure of PDAC impedes tissue perfusion, collapses blood vessels, and interferes with drug delivery, contributing to their resistance to NAC [35]. The contrast agent of CEUS is a pure blood pool agent that does not enter the extracellular space, so it can truly evaluate and quantify the vascularity of PDAC. Previous studies have confirmed the CEUS enhancement intensity was positively correlated with MVD of pancreatic cancer [36, 37] and patients with iso-enhancement PDAC live significant longer than those with hypo-enhancement pattern [14]. In our study, patients with higher Peak_tumor_/Peak_normal_ were more likely to be effective with NAC and this factor was also the most powerful biomarker for determining tumor response in the constructed nomogram, which further verified the above findings and indicated that the application of different enhancement patterns could provide a basis for risk stratification before clinical treatment in patients with PDAC. In the meanwhile, patients with a shorter TTP (≤ 25 s) were prone to present good tumor response. In the contrast, a longer TTP was associated with poor treatment response. This phenomenon may result from the special microenvironment of PDAC in which compressed and poorly functional blood vessels make it difficult for the contrast agent to enter. In brief, taller than wide shape provided the morphologic information of the lesions and CEUS features including Peak_tumor_/Peak_normal_ and TTP provided information about blood perfusion and tumor resistance to NAC, thus, the predictive nomogram based on the above factors can effectively predict the treatment response of PDAC.

Notably, there are some limitations in our study. First, this was a retrospective study conducted in a single center, selection bias may be inevitable; Second, there was a lack of information on the survival of enrolled patients, so the relationship between the independent related factors and survival was unknown; At last, there was significant difference in CA 19–9 level between the training cohort and the validation cohort and we did not validate our results in external validation cohorts, thus prospective studies conducted in multicenter need to be carried out.

## Conclusion

CEUS can give useful information about the blood perfusion of a tumor and may be useful to predict the treatment response in patients with BRPC or LAPC receiving NAC. The nomogram based on pretreatment US and CEUS has considerable potential to evaluate tumor response to NAC.

## Data Availability

The datasets used and/or analysed during the current study are available from the corresponding author on reasonable request.

## Abbreviations

CEUS: Contrast-enhanced ultrasound
PDAC: Pancreatic ductal adenocarcinoma
BRPC: Borderline resectable pancreatic cancer
LAPC: Locally advanced pancreatic cancer
NAC: Neoadjuvant chemotherapy
TTP: Time from injection of contrast agent to peak enhancement
RECIST: The Response Evaluation Criteria in Solid Tumors
CR: Complete response
PR: Partial response
SD: Stable disease
PD: Progressive disease
CA 19–9: Cancer antigen 19–9
